# Effects of *Lycium Barbarum* Aqueous and Ethanol Extracts on High-Fat-Diet Induced Oxidative Stress in Rat Liver Tissue

**DOI:** 10.3390/molecules16119116

**Published:** 2011-11-01

**Authors:** BoKang Cui, Su Liu, XiaoJun Lin, Jun Wang, ShuHong Li, QiBo Wang, ShengPing Li

**Affiliations:** 1 Department of Hepatobiliary Oncology, Cancer Center, Sun Yat-sen University, 651 Dongfeng East Road, Guangzhou 510060, Guangdong Province, China; Email: linxj@sysucc.org.cn (X.J.L.); wangj@sysucc.org.cn (J.W.); lishh@sysucc.org.cn (S.H.L.); wangqb@sysucc.org.cn (Q.B.W.); lishp@sysucc.org.cn (S.P.L.); 2 Department of Pharmocology, Guangzhou Institute for Drug Control, 23 Xizeng Road, Liwan District, Guangzhou 510160, Guangdong Province, China; Email: sliu28s@sina.com (S.L.)

**Keywords:** *Lycium barbarum*, rat, antioxidant, AST, hepatoprotection

## Abstract

This study evaluated the protective effects of aqueous extract of *Lycium barbarum* (LBAE) and ethanol extract of *Lycium barbarum* (LBEE) on blood lipid levels, serum alanine aminotransferase (ALT), aspartate aminotransferase (AST), alkaline phosphatase (ALP) activities and liver tissue antioxidant enzyme activities in rats fed a high fat diet (HF). The rats were randomly divided into seven groups of ten rats each and fed a different diet for eight weeks as follows: One group (NC group) was fed a standard diet, one group was fed a high-fat diet (HF group), one group was fed a high-fat diet and orally fed with 20 mg/kg b.w. simvastatin (HF + simvastatin group), and the other group was fed the high fat diet and orally fed with 50 mg/kg b.w. or 100 mg/kg b.w. LBAE (HF + LBAE), or 50 mg/kg b.w. or 100 mg/kg b.w. LBEE (HF + LBEE), respectively. After eight weeks, the HF diet caused deleterious metabolic effects. Rats fed the HF diet alone showed increased hepatocellular enzyme activities in plasma, a significant decline in antioxidant enzyme activities, and elevated liver lipid peroxidation indices. LBAE and LBEE administration significantly reduced liver damage and oxidative changes, and brought back the antioxidants and lipids towards normal levels. These data suggest that these antioxidants protect against toxicity parameters in HF rats.

## Abbreviations

(LBAE): aqueous extract of *Lycium barbarum*
(LBEE): ethanol extract of *Lycium barbarum*
(HF): high fat diet
(TC): total cholesterol
(TG): triglyceride
(HDL-C): high density lipoprotein cholesterol
(LDL-C): low density lipoprotein cholesterol
(ALT): alanine aminotransferase
(AST): aspartate aminotransferase
(ALP): alkaline phosphatase
(GSH-Px): glutathione peroxidase
(MDA): malondialdehyde
(GSH): glutathione
(CAT): catalase
(SOD): superoxide dismutase
(TAOC): total antioxidant capacity

## 1. Introduction

Reactive oxygen species (ROS) have detrimental effects on hepatocytes by damaging DNA, lipids, and proteins, leading to disruption in cellular homeostasis and aggravating metabolic syndrome features [1]. ROS damage cellular macromolecules causing lipid peroxidation and nucleic acid and protein alterations. Their formation is considered as a pathobiochemical mechanism involved in the initiation or progression phases of various diseases such as atherosclerosis, ischemic heart diseases, diabetes, initiation of carcinogenesis or liver diseases [2,3,4,5].

The liver plays a central role in the maintenance of systemic lipid homeostasis and is especially susceptible to ROS damage [6]. Previous studies demonstrated that chronic consumption of a high-fat diet induces obesity, liver diseases, e.g., hepatic steatosis [7,8]. Nonalcoholic fatty liver disease (NAFLD) has been identified as a risk factor for liver cancer. NAFLD is found in at least 20% of the population in developed countries and is linked with obestiy, insulin resistance and the metabolic syndrome. In some people with NAFLD, the excess fat in the liver (steatosis) causes necroinflammatory changes to the liver cell (nonalcoholic steatohepatisis [NASH]), which can progress to fibrosis and cirrhosis, and potentially, HCC. NASH affects 2–3% of the adult population in western country. Studies have shown that intake of high fat or fructose diets results in insulin resistance (IR), hepatic steatosis, excessive generation of reactive oxygen species (ROS), malfunctioning of the liver and depletion of the hepatocyte population [9]. It is noteworthy that diminished SOD, CAT, GSH-Px represent important components of the antioxidant defense enzymes [10] and increased systemic oxidative stress [11] has been found in high-fat diet induced hepatic steatosis animals.

*Lycium barbarum* (goji) fruit has become more popular over the last few years due to its public acceptance as a “super food” with highly advantageous nutritive and antioxidant properties. Concentrated extracts and infusions prepared from the berries have a history of use as ingredients in various soft or alcoholic drinks marketed [12] for their perceived anti-aging, vision, kidney and liver function benefits. In support of these traditional properties, recent studies indicate that extracts from *L. barbarum* fruit and one of its active compounds, polysaccharides (LBP), possess a range of biological activities, including effects on aging, neuroprotection, anti-fatigue/endurance, increased metabolism, glucose control in diabetics, glaucoma, anti-oxidant properties, immunomodulation, anti-tumor activity and cytoprotection [13,14,15]. There have been fewer investigations on the related *L. chinense* fruit. The focus of the investigations of the latter has been, as for *L. barbarum*, on its antioxidative properties, but more specifically in the hepatoprotection context. Free radical scavenging properties of polar extracts against the DPPH radical have been correlated with the flavonoid content [16]. A CHCl_3_-MeOH extract displayed protective activity against CCl_4_-induced hepatotoxicity. The activity of the hexane-soluble fraction of this extract was attributed to the presence of zeaxanthin and zeaxanthin dipalmitate [17]. Zeaxanthin dipalmitate reduced hepatic fibrosis induced by bile duct ligation/scission in rats at a dose of 25 mg/kg per os. The antifibrotic activity appears mediated, at least in part, by the antioxidative activity [18]. The two cerebrosides were identified as active components in the EtOAc-soluble fraction after bioactivity-guided isolation [19]. These compounds showed protective effects on rat hepatocytes exposed to CCl_4_ [20] or galactosamine [21]. Some further compounds isolated from the fruit displayed hepatoprotective activity. Study from Japan and Italy have shown that the prevalence of simple fatty liver in the general population ranges from 3–58%. Nonalcoholic fatty liver disease (NAFLD) has been identified as a risk factor for liver cancer. NFLD is found in at least 20% of the population in developed countries and is linked with obestiy, insulin resistance and the metabolic syndrome. In some people with NAFLD, the excess fat in the liver (steatosis) causes necroinflammatory changes to the liver cell (nonalcoholic steatohepatisis [NASH]), which can progress to fibrosis and cirrhosis, and potentially, HCC. NASH affects 2–3% of the adult population in western country.

In this study, we prepared aqueous and ethanol extracts of *Lycium barbarum* by traditional extraction methods. Then, hepatoprotective activities of aqueous and ethanol extracts of lycium barbarum were checked by measuring blood lipid levels, liver function tests, serum AST, ALP and ALT activities, and liver redox status, antioxidant enzyme activities, and lipid peroxidation level in rats fed a high fat diet.

## 2. Results

Livers from the high fat diet (HF) group animals showed predominant microvesicular fatty changes with focal macrovasicular fatty changes. There was no significant (*P* > 0.05) difference in rats’ body weight between groups. After eight weeks, body weights and liver index in the HF groups were heavier or higher than those of normal diet (NC) group (Table 1). These results indicated that the HF rat model had been successfully established. In all medicine-treated groups, body weight and liver indexs were significantly (*P* < 0.01) decreased compared to those of HF group. Moreover, body weight and liver indexes in the HF + LBEE group were lower than those in the HF + LBAE group. Histopathological examinations also demonstrated that LBAE and LBEE effectively stabilized the hepatocytes against HF diet by reducing the severity of hepatic lesions. Treatment with LBAE and LBEE led to lesser microvesicular fatty changes when compared with the HF group.

**Table 1:** Effect of LBAE and LBEE on body weight and liver weight of rats.

Group	Body weight		Liver index
	initial	end	
NC	80.43 + 5.32	156.63 + 12.64	4.05 + 0.32
HF	81.53 + 4.93	180.54 + 14.09 ^b^	4.78 + 0.26 ^b^
HF + simvastatin (20 mg/kg b.w.)	79.94 + 6.05	161.87 + 13.28 ^c^	4.32 + 0.31 ^c^
HF + LBAE (50 mg/kg b.w.)	80.61 + 5.37	172.6 + 13.02	4.71 + 0.29
HF + LBAE (100 mg/kg b.w.)	80.09 + 4.93	164.07 + 14.28 ^c^	4.52 + 0.32
HF + LBEE (50 mg/kg b.w.)	81.54 + 4.43	169.31 + 13.33	4.34 + 0.25 ^c^
HF + LBEE (100 mg/kg b.w.)	79.99 + 6.03	157.39 + 12.18 ^d^	4.11 + 0.27 ^c^

^b^ *P* < 0.01, compared with NC group; ^c^ *P* < 0.05, ^d^ *P* < 0.01, compared with HF group; NC: normal control.

Serum TC, TG, LDL-C levels were significantly (*P* < 0.01) elevated in the HF group compared to those of the normal diet (NC) group, but serum HDL-C level in the HF group was significantly decreased (*P* < 0.01) (Table 2). Serum levels of TC, TG and LDL-C were markedly lower in the HF + medicines (simvastatin, LBAE, LBEE) groups than those in the HF group, while HDL-C levels were significantly increased compared with those in the HF group (*P* < 0.05; *P* < 0.01). In addition, serum TC, TG, LDL-C levels in the HF + LBEE group were lower, while serum HDL-C level was higher than those in the HF + LBAE group (Table 2).

**Table 2:** Effect of LBAE and LBEE on serum TC, TG, LDL-C, HDL-C levels in rats.

Group	TC (mmol/L)	TG (mmol/L)	LDL-c (mmol/L)	HDL-c (mmol/L)
NC	2.37 + 0.14	0.69 + 0.05	0.91 + 0.04	1.22 + 0.11
HF	6.07 + 0.32 ^b^	1.29 + 0.16 ^b^	4.02 + 0.03 ^b^	0.89 + 0.07 ^b^
HF + simvastatin (20 mg/kg b.w.)	3.93 + 0.21 ^d^	0.88 + 0.03 ^d^	2.01 + 0.13 ^d^	1.13 + 0.09 ^d^
HF + LBAE (50 mg/kg b.w.)	5.63 + 0.29	1.04 + 0.08	3.31 + 0.24 ^c^	0.92 + 0.06
HF + LBAE (100 mg/kg b.w.)	4.26 + 0.19 ^d^	0.91 + 0.06 ^c^	2.84 + 0.18 ^d^	1.01 + 0.08 ^c^
HF + LBEE (50 mg/kg b.w.)	3.41 + 0.14 ^d^	0.91 + 0.08 ^d^	2.57 + 0.14 ^d^	1.09 + 0.05 ^c^
HF + LBEE (100 mg/kg b.w.)	2.29 + 0.16 ^d^	0.72 + 0.05 ^d^	1.05 + 0.09 ^d^	1.2 + 0.11 ^d^

^b^ *P* < 0.01, compared with NC group; ^c^ *P* < 0.05, ^d^ *P* < 0.01, compared with HF group; NC: normal control.

Serum AST, ALP, ALT activities were significantly (*P* < 0.01) elevated in the HF group compared to those of the normal diet (NC) group (Table 3). When rats were intragastrically fed with medicines (simvastatin, LBAE, LBEE) for eight weeks, the differences of serum AST, ALP, ALT activities between HF and HF + medicines (simvastatin, LBAE, LBEE) groups gradually increased. Serum AST, ALP, ALT activities in the HF + medicines (simvastatin, LBAE, LBEE) groups were significantly lower than those of the HF group (*P* < 0.05; *P* < 0.01). Moreover, serum AST, ALP, ALT activities in the HF + LBEE group were lower than those in the HF + LBAE group (Table 3).

**Table 3:** Effect of LBAE and LBEE on serum AST, ALP, ALT activities in rats.

Group	AST (U/L)	ALP (U/L)	ALT (U/L)
NC	182.6 + 10.52	137.42 + 10.42	35.86 + 1.43
HF	538.11 + 32.64 ^b^	497.15 + 25.62 ^b^	333.27 + 20.64 ^b^
HF + simvastatin (20 mg/kg b.w.)	273.06 + 17.59 ^d^	251.37 + 22.16 ^d^	92.15 + 5.32 ^d^
HF + LBAE (50 mg/kg b.w.)	478.21 + 30.14 ^c^	408.66 + 26.53 ^c^	281.39 + 16.42 ^c^
HF + LBAE (100 mg/kg b.w.)	370.29 + 24.1 ^d^	354.08 + 17.99 ^d^	217.58 + 17.03 ^d^
HF + LBEE (50 mg/kg b.w.)	385.32 + 19.46 ^d^	341.47 + 21.57 ^d^	194.34 + 13.29 ^d^
HF + LBEE (100 mg/kg b.w.)	227.48 + 13.82 ^d^	216.04 + 17.63 ^d^	82.14 + 5.38 ^d^

^b^ *P* < 0.01, compared with NC group; ^c^ *P* < 0.05, ^d^ *P* < 0.01, compared with HF group; NC: normal control.

The changes of liver MDA and GSH levels in rats fed HF diet for eight weeks are shown in Table 4. After eight weeks of HF feeding, liver MDA level was increased by 252.3% in the HF group, while the GSH level was decreased by 44.5% in the HF group compared with that of the normal diet (NC) group. When rats were intragastrically fed with medicines (simvastatin, LBAE, LBEE) for eight weeks, liver MDA levels of the HF + medicines (simvastatin, LBAE, LBEE) groups were significantly (*P* < 0.05; *P* < 0.01) lower, while GSH levels were significantly (*P* < 0.01) higher than in the HF group. In addition, liver MDA levels in the HF + LBEE group were lower, while liver GSH levele were higher than those in the HF + LBAE group.

**Table 4:** Effect of LBAE and LBEE on liver MDA and GSH levels in rats.

Group	MDA (nmol/mg Prot)	GSH (mg/g Prot)
NC	2.74 + 0.12	12.48 + 1.06
HF	6.92 + 0.45 ^b^	6.04 + 0.73 ^b^
HF + simvastatin (20 mg/kg b.w.)	4.35 + 0.32 ^d^	11.43 + 0.97 ^d^
HF + LBAE (50 mg/kg b.w.)	5.83 + 0.33 ^c^	8.74 + 0.77 ^d^
HF + LBAE (100 mg/kg b.w.)	4.01 + 0.19 ^d^	9.83 + 0.81 ^d^
HF + LBEE (50 mg/kg b.w.)	4.41 + 0.21 ^d^	9.91 + 0.85 ^d^
HF + LBEE (100 mg/kg b.w.)	2.93 + 0.12 ^d^	13.51 + 1.24 ^d^

^b^ *P* < 0.01, compared with NC group; ^c^ *P* < 0.05, ^d^ *P* < 0.01, compared with HF group; NC: normal control.

The antioxidant enzyme activities of the rats on the HF diet and the same diets supplemented with medicines (simvastatin, LBAE, LBEE) are shown in Table 5. The liver antioxidant enzyme activities in the HF group were significantly lower (*P* < 0.01) than those in the control group (NC). The higher liver antioxidant enzyme activities observed in the HF + medicines (simvastatin, LBAE, LBEE) groups were achieved after eight weeks of medicine administration. The TAG concentrations were not significantly (*P* > 0.05) different between the groups. In addition, liver antioxidant enzyme activities (SOD, CAT, GSH-Px and TAOC) in the HF + LBEE group were higher than those in the HF + LBAE group.

**Table 5:** Effect of LBAE and LBEE on liver SOD, CAT, GSH-Px and TAOC activities in rats.

Group	SOD	CAT	GSH-Px	TAOC
	(U/mg prot.)	(U/mg prot.)	(U/mg prot.)	(U/mg prot.)
NC	285.1 + 17.49	31.73 + 1.64	29.07 + 1.11	3.08 + 0.14
HF	121.7 + 10.83 ^b^	12.08 + 1.02 ^b^	11.38 + 0.94 ^b^	1.25 + 0.11 ^b^
HF + simvastatin (20 mg/kg b.w.)	231.6 + 18.32 ^d^	26.37 + 1.77 ^d^	28.99 + 0.99 ^d^	2.83 + 0.2 ^d^
HF + LBAE (50 mg/kg b.w.)	142.3 + 9.83 ^c^	20.15 + 1.48 ^d^	15.38 + 1.04 ^c^	1.52 + 0.11 ^c^
HF + LBAE (100 mg/kg b.w.)	179.3 + 14.01 ^d^	24.64 + 1.33 ^d^	23.15 + 1.32 ^d^	2.57 + 0.15 ^d^
HF + LBEE (50 mg/kg b.w.)	223.1 + 16.38 ^d^	25.21 + 1.09 ^d^	22.65 + 1.37 ^d^	2.62 + 0.17 ^d^
HF + LBEE (100 mg/kg b.w.)	288.4 + 24.09 ^d^	30.75 + 1.42 ^d^	30.63 + 1.54 ^d^	3.14 + 0.27 ^d^

^b^ *P* < 0.01, compared with NC group; ^c^ *P* < 0.05, ^d^ *P* < 0.01, compared with HF group; NC: normal control.

## 3. Discussion

The current study examined the effects of *Lycium barbarum* aqueous and ethanol extracts on oxidative stress and antioxidant enzymes in the liver of rats fed a high-fat diet. In this study, the high-fat diet animals displayed significantly increased body weight and liver indexes compared to those in the normal diet group, but the body weight and liver indexes in the groups administrated *Lycium barbarum* aqueous and ethanol extract were significantly decreased compared to those in the HF group. The effect was dose-dependent. This indicated that LBAE and LBEE can reduce accumulation of visceral fat in HF rats.

The liver regulates systemic lipid homeostasis, which is involved in the redistribution of lipoproteins, triacylglycerol for storage and utilization by peripheral tissues [22]. High plasma TC is suggested as an important factor for the development of inflammation in mice [23]. In the present study, high fat diet reduced plasma HDL-C and elevated LDL-C, TG, TC levels in the HF rats. Both aqueous and ethanol extracts of *Lycium barbarum* had a strong hypotriglyceridemic and hypocholesterolemic effects in plasma of rats with a reduction of plasma LDL-C levels and an increase in HDL-C levels. These findings seem to be in accordance with the results of Yang *et al.* [24], who reported significant increases in serum TG, HDL and LDL levels in rats fed a high-fat diet. Furthermore, the aqueous and ethanol extracts of *Lycium barbarum* both markedly decreased plasma TC and TG levels in HF rats. These results suggest that LBAE and LBEE could be helpful in decreasing the incidence of steatohepatitis diseases through a reduction in TC and LDL-C and an increase in HDL-C.

On the other hand, the liver is a major target organ for thyroid hormone with important biological and medical implications [25,26]. Clinical diagnosis of disease and damage to the structural integrity of the liver is commonly assessed by monitoring the status of serum AST, ALP and ALT activities, which are sensitive serological indicators of liver toxicity [27]. Higher activities of these enzymes in serum have been found in response to oxidative stress induced by high fat diets [7,8]. In our study these parameters were significantly enhanced by the high fat diet, suggesting that excessive fat intake might cause critical injury to the organ due to the over-production of free radicals and ROS, which exert deleterious effects on liver. Decreased serum AST, ALP and ALT activities were observed in the LBAE- and LBEE-treated rats, suggesting that aqueous and ethanol extracts of *Lycium barbarum* can reverse liver toxicity induced by high fat diets.

It is well known that oxidative stress is associated with metabolic syndrome and is related to the accumulation of visceral fat [28]. Fardet *et al.* [29] suggested that rats in diet-induced obesity models showed an increase in the levels of oxidative stress in their liver and that oxidative stress can result from the excessive production of reactive oxygen species and/or deficient anti-oxidant capacity [29,30].

In the present study, we measured the amount of MDA in livers of HF fed rats to get an indication of the amount of *in vivo* oxidative stress. The measurement results reveal that MDA levels were significantly higher in livers of rats fed a HF diet compared with those fed a normal diet, which may indeed indicate an increased amount of oxidative stress in the HF fed rats. The biological antioxidant defense system is an integrated array of enzymes and antioxidants. These include GSH, a substrate for GSH-peroxidase, SOD that catalysis the destruction of O_2_^−^ by dismutation and hydrogen peroxide formation, catalase and GSH-peroxidase that catalyze the conversion of hydrogen peroxide to water [31]. GSH is required to maintain the normal reduced state and to counteract the deleterious effects of oxidative stress. During the reduction of hydrogen peroxide, GSH is oxidized to GSSG. When GSSG levels are enhanced, the GSH-reductase activity was activated to convert GSSG in GSH.

Significant changes were observed in the antioxidant systems, comparing the HF and normal diet groups, indicating that ROS generation was enhanced by high-fat diet intake. In fact, there is a growing awareness that the use of oxygen is vital for oxidative phosphorylation by the electron transport system, and alterations in food constituents, as occur in high-fat diet intake, result in higher ROS production, thus inducing oxidative stress and lipid hydroperoxide formation [32,33]. Some previous studies have reported that other plant extracts that have antioxidant properties can protect the liver from various toxicants [34,35,36,37]. Because of the high production yield of *Lycium barbarum* and strong free radical scavenging activity, its aqueous and ethanol extract are superior to other plant extracts in protecting the liver from toxicants. A notable finding of this study was that both aqueous and ethanol extracts of *Lycium barbarum* intake had distinct effects on hepatic oxidative stress markers, and eight weeks of LBAE and LBEE feeding can significantly reduce liver MDA levels, and enhance SOD, CAT, GSH-Px and TAOC activities.

## 4. Material and Method

### 4.1. Plant Material and Extraction

The fruits of *Lycium barbarum* was obtained from a herb shop in Guangzhou in February 2010. The plant was identified by Prof. Dr. CM Wang, Department of Pharmacology, by comparing with the voucher specimen deposited in the herbarium of the Faculty of Pharmacy, serial number (LB) *Lycium barbarum* 201102032. The fruits was dried at room temperature. By ultraviolet-visible absorption spectroscopy, it can be found that *Lycium barbarum* fruits contain significant levels of antioxidant material (anthocyanin – 11.6 mg/g) and total polyphenols (1.54 mg/g). The dried fruits (500 g) were put in 3 L of hot water (100 °C) and macerated during 4 h following the traditional method. The solution obtained after filtration with Whatman No. 3 filter paper, was lyophilized and gave 147 g (29.4%) of *Lycium barbarum* aqueous extract. The dried fruits (500 g) were also put in 3 L of ethanol and extracted for 3 h at 70 °C. At the end of the extraction time, the solution obtained after filtration with Whatman No. 3 filter paper, was evaporated under reduced pressure at 35 °C; the w/w yield from dry material (*Lycium barbarum* ethanol extract) was 37 g (7.4%).

### 4.2. Animals and Dietary Treatment

Seventy male Wistar rats (2 month old) weighing 245 ± 10 g were housed in stainless steel cages in a room with controlled lighting (12-h light:dark cycle), constant temperature (24 °C) and relative humidity (60%). The animals were randomly divided into seven groups of ten rats each and fed a different diet for eight weeks as follows: one group (NC group) was fed a standard diet, one group was fed a high-fat diet (HF group), one group was fed a high-fat diet (HF group) and orally fed with 20 mg/kg b.w. simvastatin (HF + simvastatin group), and the other groups were fed the high fat diet and orally fed with 50 mg/kg b.w. or 100 mg/kg b.w. LBAE (HF + LBAE), or 50 mg/kg b.w. or 100 mg/kg b.w. LBEE (HF + LBEE), respectively. The LBAE and LBEE dosage was selected based on a previous test wherein LBAE or LBEE administered at 100 mg/kg b.w./day for eight weeks did not cause any toxic symptoms in rats nor affected food intake or body weight gain. The 20–100 mg/kg BW used in these studies is equal to 3.24–16.21 mg/kg BW for human. The composition of diets is detailed in Table 6. Diets and tap water were freely available. The animals were weighed weekly. We followed the general guidelines on the use of living animals in scientific investigations of Sun Yat-sen University.

**Table 6:** Composition of the experimental diets (g/kg) fed to rats [38].

Ingredients	ND	HFD
Casein	200	200
dl-Methionine	3	3
Corn starch	150	111
Sucrose	500	370
Cellulose	50	50
Corn oil	50	30
Lard	–	170
Mineral mixture ^a^	35	42
Vitamin mixture ^b^	10	12
Choline bitartrate	2	2
Cholesterol	–	10
tert-Butylhydroquinone ^c^	0.01	0.04
Fat, % kJ	11.5	40.0
Total energy, kJ/kg diet	16,439	19,315

^a^ Mineral mixture for AIN-76A rodent diet; ^b^ Vitamin mixture for AIN-76A rodent diet; ^c^ Antioxidant added at 0.01 g/50 g lipid.

One hour after extract administration, rats were weighed and blood was collected from abdominal aorta into dried tubes. The collected blood samples were centrifuged at 5,000 rpm for 10 min to separate the plasma, which was stored at −20 °C until further analysis. After sacrifice, the livers were frozen immediately under liquid N_2_, weighed separately, and kept at −80 °C until the analysis.

### 4.3. Histopathological Analysis

Histology of liver was studied using hematoxylin and eosin (H and E) and oil red O staining. A portion of the liver was fixed in 10% buffered formalin, dehydrated in graded (50–100%) alcohol embedded in paraffin. Thin sections (4–5 μm) were cut and stained with H and E. For oil red O staining, frozen liver sample was processed using cryostat and then fixed and stained.

### 4.4. Biochemical Analysis

Serum levels of total cholesterol (TC), triglyceride (TG), high density lipoprotein cholesterol (HDL-C) and low density lipoprotein cholesterol (LDL-C) were measured using ELISA kits (Bionewtrans Pharmaceutical Biotechnology Co., Ltd., Franklin, MA, USA). Serum levels of alanine aminotransferase (ALT), aspartate aminotransferase (AST) and alkaline phosphatase (ALP) were measured using commercial kits (Bayer Diagnostics, Tarrytown, NY, USA). Lipid peroxidation was measured according to MDA diagnostic kits. In brief, samples were mixed with TBA reagent. The reaction mixtures were placed in a boiling water bath for 40 min and centrifuged at 4,000 rpm for 10 min. The absorbance of the supernatant was measured at 532 nm. The results were expressed as nmol/mg protein.

The tissue GSH concentrations were measured using the method described by Beutler *et al.* [39]. Briefly, fresh supernatant (0.2 mL) was added to distilled water (1.8 mL). Three milliliters of the precipitating solution (1.67 g metaphosphoric acid, 0.2 g EDTA and 30 g NaCl in 100 mL distilled water) was mixed with haemolysate. The mixture was allowed to stand for approximately 5 min and then filtered (Whatman No. 42). Two milliliters of filtrate was taken and added into another tube, and then the phosphate solution (8 mL, 0.3 M disodium phosphate) and DTNB (1 mL) were added. A blank was prepared with phosphate solution (8 mL), diluted precipitating solution (2 mL, three parts to two parts distilled water), and DTNB reagent (1 mL). A standard solution of glutathione was also prepared (40 mg/100 mL). The optical density was measured at 412 nm in the spectrophotometer.

The superoxide dismutase activity was determined using a method of Asada *et al.* [40]. SOD activity was assayed by measuring its ability to inhibit the photoreduction of NBT (nim). One millilitre of homogenate supernatant is combined with 50 mm phosphate buffer (pH 7.8), 39 mM methionine, 2.6 mM NBT, and 2.7 mM EDTA. To obtain a concentration of 0.26 mM, riboflavin was added as the last, and switching on the light started the reaction. The changes in absorbance at 560 nm were recorded after 20 min. One unit of SOD is defined as the amount that inhibits the NBT reaction by 50%. Specific activity was defined as U/mg protein.

CAT activity was determined using the method described by Beutler [41]. Briefly, 1 M Tris-HCl, 5 mM EDTA (pH 8), 10 mM H_2_O_2_ and H_2_O were mixed and the rate of H_2_O_2_ consumption at 240 nm and 37 °C was used for quantitative determination of CAT activity (20 μL of 95% ethanol was added to 1 mL of hemolysate to break down complex of catalase and H_2_O_2_). The amount of enzyme capable of catalyzing the degradation of 1 μmol of H_2_O_2_ per minute was defined as 1 U.

Activity of glutathione peroxidase (GSH-Px) was determined according to the method of Lawrence and Burk [42]. The assay mixture consisted of 75 mM phosphate buffer (2.0 mL, pH 7.0), 60 mM glutathione (50 μL), 30 units/mL glutathione reductase (0.1 mL), 15 mM EDTA (0.1 mL), 3 mM NADPH (0.1 mL) and the appropriate amount of tissue supernatant to a final volume of 3.0 mL. The reaction was started by the addition of 7.5 mM H_2_O_2_ (0.1 mL). The rate of change of absorbance during the conversion of NADPH to NADP^+^ was recorded spectrophotometrically at 340 nm for 3 min. GSH-Px activity for tissues was expressed as μmoles of NADPH oxidized to NADP^+^ min^−1^ mg^−1^ protein. Total antioxidant capacity was determined using commercial kits assay (Shanghai BoYun Biotechnology Ltd, Shanghai, China) according to the method of Strube *et al.* [43].

### 4.5. Statistical Analysis

The data were expressed as means ± SD based on the indicated number in the experiment. All analyses of data were done with the Statistical Package for Social Sciences (version 11.0; SPSS, Chicago, IL, USA). The results were analyzed using 1-way analysis of variance followed by Student *t* test for comparison between different treatment groups. Statistical significance was set at *P* < 0.05.

## 5. Conclusions

On the basis of the results obtained in the present study, we conclude that the aqueous and ethanol extracts of *Lycium barbarum* can enhance liver tissue antioxidant enzyme activities, and reduce lipid peroxidation in rats fed a high fat diet. Therefore, both aqueous and ethanol extracts of *Lycium barbarum* have strong antioxidant activities and can prevent or reduce the effects of HFD on several parameters of toxicity in HF rats. Moreover, ethanol extracts of *Lycium barbarum* display stronger antioxidant and hepatoprotective effects than aqueous extract of *Lycium barbarum*. The polyphenolic constituents may be responsible for the inhibition of lipid peroxidation and enhance the antioxidant activities of ethanol extract of *Lycium barbarum*. Further studies are needed to isolate the active components from this plant.
